# Development of Tea Seed Oil Nanostructured Lipid Carriers and In Vitro Studies on Their Applications in Inducing Human Hair Growth

**DOI:** 10.3390/pharmaceutics14050984

**Published:** 2022-05-04

**Authors:** Pornthida Riangjanapatee, Mattaka Khongkow, Alongkot Treetong, Onuma Unger, Chutikorn Phungbun, Supatchaya Jaemsai, Chatchaya Bootsiri, Siriporn Okonogi

**Affiliations:** 1 National Nanotechnology Center (NANOTEC), National Science and Technology Development Agency (NSTDA), Pathum Thani 12120, Thailand; mattaka@nanotec.or.th (M.K.); alongkot@nanotec.or.th (A.T.); onuma.nanomail@gmail.com (O.U.); chutikorn@nanotec.or.th (C.P.); supatchaya.jae@nanotec.or.th (S.J.); fafah.siri@hotmail.com (C.B.); 2 Department of Pharmaceutical Sciences, Faculty of Pharmacy, Chiang Mai University, Chiang Mai 50200, Thailand; okng2000@gmail.com; 3 Research Center of Pharmaceutical Nanotechnology, Chiang Mai University, Chiang Mai 50200, Thailand

**Keywords:** tea seed oil, nanostructured lipid carriers, human follicle dermal papilla, natural ingredient, spreadability, texture analysis, hair serum

## Abstract

Synthetic drugs used to treat hair loss cause many side-effects. Natural tea seed oil possesses many activities that can suppress hair loss. However, it is oily and sticky in direct application. In this study, tea seed oil loaded nanostructured lipid carriers (NLC) using Tween 80 (NLC-T), Varisoft 442 (NLC-V), and a combination of both surfactants (NLC-C) was developed. The obtained nanoformulations showed spherical particles in the size range 130–430 nm. Particle size and size distribution of NLC-C and NLC-T after storage at 4, 25, and 40 °C for 90 days were unchanged, indicating their excellent stability. The pH of NLC-T, NLC-V, and NLC-C throughout 90 days remained at 3, 4, and 3.7, respectively. NLC-C showed significantly greater nontoxicity and growth-stimulating effect on human follicle dermal papilla (HFDP) cells than the intact oil. NLC-T and NLC-V could not stimulate cell growth and showed high cytotoxicity. NLC-C showed melting point at 52 ± 0.02 °C and its entrapment efficiency was 96.26 ± 2.26%. The prepared hair serum containing NLC-C showed better spreading throughout the formulation than that containing the intact oil. Using 5% NLC-C showed a 78.8% reduction in firmness of the hair serum while enhancing diffusion efficiency by reducing shear forces up to 81.4%. In conclusion, the developed NLC-C of tea seed oil is an effective alternative in stimulating hair growth. Hair serum containing NLC-C obviously reduces sticky, oily, and greasy feeling after use.

## 1. Introduction

Hair is an important physical aspect of human beings. Hair can protect the scalp from the severe environment of heat, cold, and radiation, as well as serving the beauty of the body. Therefore, loss of hair can result in distress and psychological problems. Hair loss (alopecia) affects both men and women of all ages and often has significantly impacts on social and psychological wellbeing. Androgenetic alopecia is the most common cause of hair loss in humans [1,2]. Minoxidil and finasteride are the only two synthetic drugs which the US Food and Drug Administration (FDA) approved to use as a treatment of androgenetic alopecia [3]. Common adverse effects of minoxidil include redness, dryness, scalp irritation, itchiness, and contact dermatitis [4,5]. It was previously reported that 6% contact dermatitis was found in 393 androgenetic alopecia participants using daily 5% topical minoxidil solution at the scalp target area [6]. In 2015, severe side-effects including testicular pain, impotence, abnormal sexual functions, myalgia, gynecomastia, and abnormal ejaculation were reported after using finasteride [7]. As there are only these two synthetic drugs currently available in the market and both have adverse effects, therefore, a search for new ingredients, particularly from natural sources to treat hair loss, remains a challenging problem.

The hormonal system problems, endocrine disorder, certain drugs, nutritional deficiencies (protein, vitamins, and minerals), and infections are factors that retard hair growth. Subsequently, they often result in hair loss [8,9]. Emotional distress is also one of the most important factors in hair growth and often causes hair loss [10]. It is widely accepted that stress can cause response reactions in cells of a living organism, namely, free radical oxidation. Therefore, a substance having antioxidant property can support hair growth by terminating this free radical oxidation. In addition, a deep inflammatory process around the follicle accelerates the shedding hair and retards hair growth [11].

Tea seed oil is a non-volatile oil extracted from seeds of tea (*Camellia oleifera*), a plant in the family Theaceae. It is a yellow viscous liquid, sticky, and gooey. Tea seed oil has high content of compounds having antioxidant activity and is a rich source of emollients for skin care and minimizing signs of aging [12]. It has smoothing properties and is considered non-comedogenic. The important chemical composition of tea seed oil is similar to olive oil, which contains a low proportion of saturated fatty acids and high unsaturated fatty acids including omega 9, omega 6, and omega-3 acids. In addition, it contains vitamins A, B, C, D, and E, and antioxidants such as catechin. Tea seed oil contains important fatty acids and is commonly used as an emulsifier, emollient, and lubricant in creams and lotions in cosmetics and skin cleansing products [13]. It increases the permeability of active ingredients and brings low-polar substances into human skin [14]. The predominant fatty acid (75–81%) in tea seed oil is oleic acid, which has high nutritional value in preventing oxidation and can inhibit Gram-positive bacteria [15,16]. The other fatty acids, even in low quantity, such as stearic acid, linoleic acid, or omega 6, can reduce dandruff, stimulate hair growth, moisturize the skin and nails, reduce oiliness and inflammation, and soften the skin [17,18,19]. Plant extracts containing linoleic acid have anti-inflammatory effect and are used as an active substance in acne products [20]. 

Previously, tea seed oil was reported to have antioxidant properties [21,22,23]. As tea seed oil can terminate free radical oxidation, it therefore supports hair growth. There are several studies that have investigated the mechanism of oleic acid, which is the major compound in tea seed oil, in promoting hair growth [24,25,26]. It is concluded that a mechanism of oleic acid on supporting hair growth is to accelerate anagen production of hair follicles and is related to the activation of the Wnt/β-catenin signal pathway. From the biological properties of tea seed oil mentioned above, it is clearly seen that tea seed oil is a great candidate suitable for stimulation of hair growth. However, the stability of the oil during storage could seriously shorten its shelf life. In addition, direct application of the oil can cause a sticky and oily feeling after use. Nanoencapsulation technology is used to protect and enhance the stability of active ingredients in pharmaceutical and medical applications [27,28]. Among them, the lipid-based colloidal delivery system, such as solid lipid nanoparticle, microemulsion, nanoemulsion, and nanostructured lipid carrier (NLC), has received great attention as it reveals a high biocompatibility, biodegradability, and good shelf life [29,30,31]. In addition, during the past decades, NLC has attracted increasing attention for a cutaneous delivery system because of its high efficiency in increasing bioavailability of the entrapped active compounds. Importantly, NLC is able to protect chemically labile actives against degradation [32,33]. It is also readily for a large-scale production. In the context of dermal drug delivery, penetration of active compounds into the human skin depends strongly on skin hydration, which can be affected by occlusive compounds [34]. NLC itself has an occlusion property, which leads to increased skin hydration due to reduced water loss. Several reviews have reported numerous benefits of NLC systems having high loading capacity of active compounds [35], as well as minimum drug expulsion during storage. Therefore, the use of NLC for enhancing stability of tea seed oil was considered in the present study. To the best of our knowledge, this is the first reported study on an NLC preparation containing tea seed oil for the treatment of hair loss. The resulting NLC system is expected to be a promising product with new ingredients extracted from natural sources to replace the toxic synthetic available drugs, minoxidil and finasteride. The particles of the obtained NLC were investigated for their physicochemical properties including morphology, size, size distribution, and stability. The cytotoxicity and cell proliferation of the product were also investigated. In addition, hair serum containing the most suitable NLC was prepared, and its texture was compared with that containing the intact oil.

## 2. Materials and Methods

### 2.1. Materials

Tea seed oil was provided by the Chaipattana Foundation. Olivem 1000 (cetearyl olivate, sorbitan olivate) was purchased from Hallstar, Suzhou, China. Varisoft 442 100P (quaternium-18) was provided by Evonik, Essen, Germany. Ammonium Acryloyldimethyltaurate/VP Copolymer was purchased from Clariant AG, Muttenz, Switzerland. Phenoxyethanol, chlorphenesin, trolox, and 2,2-diphenyl-1-picrylhydrazyl (DPPH) were purchased from Sigma–Aldrich (St. Louis, MO, USA). Ultra-pure water was prepared using a Milli-Q Plus system (Millipore1, Schwalbach, Germany). Tween 80 was from Panreac, Barcelona, Spain. Ethanol, dimethyl sulfoxide (DMSO), glycerin, minoxidil, and asiaticoside were from Merck, Darmstadt, Germany. Human follicle dermal papilla (HFDP) (C-12071, promocell) cell lines and Follicle Dermal Papilla Cell Growth Medium (C-26501, promocell) were purchased from the American Type Culture Collection (Manassas, VA, USA). Hydrogen peroxide solution 30% stabilized ACS was purchased from VWR chemical BDH, Radnor, PA, USA. All other chemicals were of the highest grade available.

### 2.2. DPPH Scavenging Activity and Effects on HFDP Cells

In this experiment, antioxidant activity of tea seed oil on scavenging DPPH free radicals was firstly determined. Tea seed oil ethanolic solution was prepared by dissolving in ethanol to yield a concentration of 0.1 mg/mL. DPPH was dissolved in ethanol and mixed with the ethanolic oil solution. The solution was adjusted to a final DPPH concentration of 100 µM using ethanol. The mixture was shaken vigorously and left to stand for 30 min in the dark at room temperature. The amount of DPPH remaining in each period of stand was determined spectrophotometrically at 540 nm using a microtiter plate reader (SpectroStar Nano (Allmendgrün, Germany)). All measurement was performed in triplicate. The antioxidant of tea seed oil was expressed as Trolox equivalent antioxidant capacity (TEAC) value, which was obtained by comparing the absorbance change at 540 nm in a reaction mixture containing an oil sample with that containing Trolox. The antioxidant activity of tea seed oil was subsequently confirmed in HFDP cells.

In HFDP cells, the antioxidant activity of the samples against free radical induction by hydrogen peroxide (H_2_O_2_) was investigated. As HFDP cells are commercially available, there is no need to gain ethical approval. The cells were grown in Dulbecco’s modified Eagle’s medium (DMEM) supplemented with 10% fetal bovine serum (FBS), 1% L-glutamine, 100 U/mL penicillin, and 100 µg/mL streptomycin in a humidified atmosphere of 5% CO_2_, 95% air at 37 °C. The cells, with a concentration of 1 × 10^6^ cell/mL, were cultured in each well of a 96-well plate for 24 h. After incubation, the cells were treated with the samples at concentration 0–2000 µg/mL for an additional 24 h. After removal of the sample solution, the cells were incubated with hydrogen peroxide (H_2_O_2_) at various concentrations for 2 h. Then, 5 mg/mL MTT was added. After 4 h incubation, MTT solution was discarded and 100% DMSO 100 µL was filled into each well. Viable cells were determined by measuring the absorbance at 572 nm. Data were presented as the mean values ± standard deviation calculated from triplicate experiments.

### 2.3. Cytotoxicity and Cell Growth-Stimulating Effects

Cytotoxicity and cell growth-stimulating effects of tea seed oil were compared with those of minoxidil and asiaticoside. The control group was a specimen without treatment with the tested samples. The samples were determined by MTT assay [36]. MTT assay is a method which is generally used to investigate the number of viable cells. Therefore, this assay can be used for proliferation and cytotoxicity studies [37]. The MTT result showed the percentage of cell viability, and a control was set as 100%. The percentages of cell viability of the samples were compared with the control. The samples which showed percent cell viability lower than the control indicated their toxicity to the cells and cause of cell death. On the other hand, the samples which showed percent cell viability higher than the control indicated their increasing the number of cells or promoting cell growth. The same procedure as an antioxidant test in HFDP cells was carried out. Tea seed oil, minoxidil, and asiaticoside were tested at concentrations 0–8000 µg/mL. NLC formulations and their blank were tested at concentrations 0–2000 µg/mL. 

### 2.4. Preparation of NLC and Hair Serum 

Three NLC formulations containing tea seed oil by using Tween 80 (NLC-T), Varisoft 442 (NLC-V), and a combination of Tween 80 and Varisoft 442 (NLC-C) based on the composition listed in Table 1 were prepared according to the high-speed homogenization technique at hot temperatures. In brief, tea seed oil was dissolved in the oil phase of molten Olivem 1000. For the aqueous phase, the surfactant system containing Tween 80 or Varisoft 442 100P, or a combination of both surfactants was dissolved in water. The oil phase and the aqueous phase were separately heated to 75 °C. Then, they were combined and vigorously stirred using a Heidolph Silent Crusher M (Schwabach, Germany) at 18,000 rpm high-speed stirring for 5 min to obtain a hot pre-emulsion. After that, the lipid dispersion was abruptly cooled down by putting in the ice bath (4 °C). This process is to solidify the lipid droplet phase and to obtain the aqueous NLC dispersions.

Hair serums were prepared by heating ultra-pure water at 40 °C. Ammonium acryloyldimethyltaurate/VP copolymer was added and continuously stirred. Then, glycerin, phenoxyethanol, and chlorphenesin were subsequently added. The obtained mixtures were cooled to ambient temperature.

### 2.5. Particle Characterization

Morphological structure and surface properties of the prepared NLC nanoparticles were investigated using a transmission electron microscope (TEM) (JEM-1200EX, JEOL, Tokyo, Japan) and atomic force microscope (AFM) (SPA 3800N, SEIKO, Tokyo, Japan), respectively. A TEM sample was prepared by dropping 50-fold diluted oil with double-distilled water onto a 400-mesh copper grid coated with carbon film and followed by negative staining with 1% uranyl acetate. The samples were then dried in a desiccator until analysis. The AFM was operated in a tapping mode, using high-resonant-frequency (F0 = 129 kHz) pyramidal cantilevers with silicon probes having force constants of 20 N/m. Scan speed was set at 2 Hz. The samples were diluted 10 times with distilled water and then dropped onto freshly cleaved mica plates, followed by vacuum drying for 24 h at 25 °C. The size of the NLC particles can be estimated using bar scales on both TEM and AFM images.

### 2.6. Stability Test

Stability of NLC nanoparticles (NLC-T, NLC-V, NLC-C) was tested by keeping samples at 4 °C, 25 °C, or 40 °C for 3 months. For temperature-fluctuated condition, the NLC samples were kept at 4 °C for 48 h and 45 °C for 48 h, which was defined as one cycle. The study was conducted in a total of eight cycles. Averaged particle size, size distribution, and pH were evaluated on day 0 (D0) and 1, 2, and 3 months (1 M, 2 M, 3 M, respectively) of storage. The particle size and size distribution were determined using a photon correlation spectrophotometer (PCS) (Zetasizer 4, Malvern Instruments, Herrenberg, Germany). A volume of 100 µL was diluted with 900 µL deionized water in order to eliminate multiple scattering. The samples were measured at an angle of 273°. The average diameter was calculated according to Stokes–Einstein after a curve fitting of the correlation function was performed [38]. pH value of the samples was measured using a pH meter (Mettler Toledo).

### 2.7. Thermal Behavior Investigation

Thermal behavior of the samples was assessed using a differential scanning calorimeter (DSC) (DSC-60A Plus DSC) (Shimadzu, Kyoto, Japan). The samples were accurately weighed and placed in aluminum pans. After that, the pan was sealed with a lid. Al_2_O_3_ was used as the reference. In the scanning process, a heating rate of 5 °C was applied in the temperature range from 20 to 80 °C under nitrogen gas purged at 40 mL/min.

### 2.8. Chemical Analysis of Tea Seed Oil

Tea seed oil was analyzed using gas chromatography (GC) (GC 7890A-5975C) Agilent Technologies. The analytical column was DB-5HT (30 m × 0.250 mm × 0.10 µm). The carrier gas was helium with constant flow rate of 1 mL/min and the split ratio of 50:1. The oven temperature was programed at 40 °C and hold for 5 min, ramping from 40 °C to 200 °C at 10 °C/min, and holding at 250 °C for 15 min. The injection and MS detector source temperature were maintained at 250 and 230 °C, respectively. The MS was operated in the electron ionization mode with ionization energy of 70 eV. The data acquisition was elaborated with Chemstation software using Wiley (10^th^ Edition) with NIST Mass Spectral Library (NIST 2014). The chromatographic peaks were based on the comparison of their mass spectra fragmentation patterns and retention time with NIST library and oleic acid standard. Method linearity was checked by establishing external calibration curves. A stock solution of oleic acid standard was prepared in hexane and diluted to five different concentration levels, ranging from 0.5 mg/mL to 10.0 mg/mL. Each level was analyzed in triplicate.

### 2.9. Drug Loading Efficiency

The amount of tea seed oil in NLC was calculated by determining the amount of encapsulated oil using the ultrafiltration technique. An exact amount of 1.0 mL of tea seed oil-loaded NLC colloidal solution was placed in the upper chamber of a centrifuge tube matched with an ultrafilter (Amicon ultra, Millipore Co, Billerica, MA, USA, MWCO 3 kDa) and centrifuged at 5000× *g* for 60 min. This collected NLC was dissolved with 5 mL hexane and determined by GC. Entrapment efficiency (EE) value is defined as the percentage of drug incorporated into the lipid nanoparticles relative to the total drug added. The EE value can be calculated using Equation (1) [39].

(1) $$\mathrm{EE}\;\left(\%\right)=\frac{\mathrm{Weight}\;\mathrm{of}\;\mathrm{encapsulated}\;\mathrm{drug}}{\mathrm{Total}\;\mathrm{weight}\;\mathrm{of}\;\mathrm{drug}\;\mathrm{in}\;\mathrm{NLC}}\times 100.$$

### 2.10. Texture Analysis

The measurement of texture of the prepared hair serum was performed and interpreted using a texture analyzer (Stable Micro Systems, TA.XT Plus, Godalming, UK) under the following conditions: 50 g of applied force and 5 kg of load cell. The sample was poured into a conical sample holder until it was completely full. A stirring rod was used to smooth the surface of the sample. Once the test was commenced, the cone probe proceeded and penetrated the sample to a depth of 2 mm above the sample holder surfaces. In this test, the probe moved 23 mm from the starting point at a rate of 3 mm/s. The instrument measured the applied force at different times. The relationship between force and time was shown in a graph called force–time curve. Firmness was calculated from the value of maximum force to shear. The work of shearing the sample (work of shear) could be obtained from the area under the graph. The samples were analyzed in triplicate.

### 2.11. Statistical Analysis

Data were presented as the mean ± S.D. (n = 3). Statistical analysis was conducted by Student’s *t*-test or Analysis of Variance (ANOVA) using Graphpad version 7.0 to determine statistical significance at *p values of < 0.05, **p values of <0.01, ***p values of <0.005) with Tukey post hoc test.

## 3. Results and Discussion

### 3.1. Antioxidant Activity, Cytotoxicity, and Cell Growth-Stimulating Effects of Tea Seed Oil

**Figure 1:**
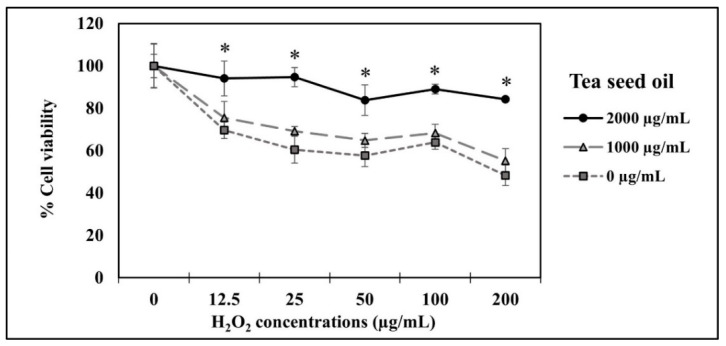
Effects of tea seed oil on protecting HFDP cells from free radicals induced by H_2_O_2._ (* indicates statistical significance at *p* < 0.05).

Hair follicles of any hair type have a unique life cycle. The regulation of the hair cycle is complicated and involves several factors including cytokine imbalance and oxidative stress [40,41]. It is known that oxidative stress is an imbalance between free radicals and endogenous antioxidants. Free radicals can activate a number of molecular pathways that generate inflammatory cytokines, such as interleukin 1 beta (IL-1β), interleukin 6 (IL-6), and tumor necrosis factor alpha (TNF-α) [42,43]. Therefore, excessive free radicals can cause abnormal hair follicle cycling and subsequent hair loss. Ingredients with high antioxidant activity should be able to protect against hair loss. In the present study, the antioxidant activity of tea seed oil was preliminarily studied in scavenging DPPH free radicals. The result indicates that tea seed oil has high antioxidant activity, with a TEAC of 634.206 ± 5.215 µM/mg oil. Subsequently, the antioxidant activity of tea seed oil on free radicals was analyzed in H_2_O_2_-induced HFDP cells. The percentages of viable cells were compared between the H_2_O_2_-treated and nontreated groups. The results as shown in Figure 1 reveal that the percentage of viable cells decreases with increasing H_2_O_2_ concentrations. Interestingly, the percentage of viable cells increases with increasing tea seed oil concentration. It is found that HFDP cells containing 2000 µg/mL of tea seed oil have a survival percentage greater than 90% at all tested concentrations of H_2_O_2_, which is significantly higher than that without tea seed oil. The treatment of tea seed oil at a concentration of 1000 µg/mL resulted in a survival rate of HFDP cells at approximately 60%, and 50%, respectively. These results obviously indicate that tea seed oil is effective in protecting HFDP cells from free radicals. Our result is in good agreement with other previous reports on potentially antioxidant activity of tea seed oil. For example, tea seed oil can decrease H_2_O_2_-mediated formation of free radical species in red blood cells [23] and it can protect the liver against CCl_4_-induced oxidative damage in rats via increasing the activities of glutathione peroxidase, glutathione reductase, and glutathione S transferase [22].

**Figure 2:**
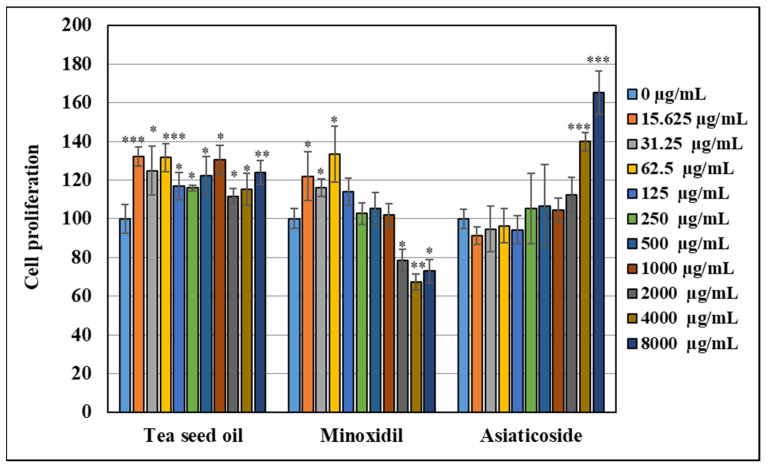
Cytotoxicity and cell proliferation of HFDP cells treated with tea seed oil compared to minoxidil and asiaticoside. (* indicates statistical significance at *p* < 0.05, ** indicates statistical significance at *p* < 0.01, *** indicates statistical significance at *p* < 0.005).

Minoxidil is FDA-approved as a treatment for androgenetic alopecia [2,44]. It has been shown that a mechanism of minoxidil inducing hair growth is via stimulation of vascular endothelial growth factor (VEGF), which plays a role in promoting angiogenesis as well as influencing cell survival and proliferation [45]. This compound was selected to compare the activity with tea seed oil in this study. In addition, it was previously reported that *Centella asiatica* enlarged follicular size and prolonged the growth phase as well as inhibited the activity of 5α-reductase that caused hair loss [46]. The ethanolic extract of this plant can promote hair growth via enhancing VEGF expression in HFDP cells. The major active compound of *C. asiatica* is asiaticoside [47]. Therefore, this compound was also selected to compare the activity with tea seed oil in this study. The results are shown in Figure 2. It is found that minoxidil at a concentration range of 15–1000 µg/mL can stimulate the growth of HFDP. At higher concentrations, e.g., in the range of 2000–8000 µg/mL, minoxidil is toxic to HFDP cells and decreases cell proliferation. Our results show that only high doses of asiaticoside, such as 4000–8000 µg/mL, can stimulate HFDP cell growth. Tea seed oil shows significantly higher cell growth-stimulating effect from a low concentration of 15 µg/mL to a high concentration of 8000 µg/mL, in a concentration-independent manner. These results obviously indicate that tea seed oil possesses a significantly higher growth-stimulating effect on HFDP cells, particularly at low concentration, compared to minoxidil and asiaticoside. To the best of our knowledge, there are no reports on using tea seed oil for hair growth stimulation. Previous studies showed the effect of oleic acid on hair growth promotion by stimulating dermal papilla growth [48,49] and reducing the expression of 5-α reductase [50]. As oleic acid is the major active compound in tea seed oil, it is considered that the hair growth-stimulating effect of tea seed oil is mainly due to oleic acid present in the oil. The mechanism of promoting hair growth of oleic acid was previously proposed to be due to the acceleration of hair follicles into the anagen and activation of the Wnt/β-catenin signal pathway [25].

### 3.2. Preparation of NLC

Different types of lipids (e.g., solid lipid, liquid lipid) create different types of nanoparticles. The use of liquid lipids or oils alone will form a nano-emulsion formulation [51]. However, using liquid lipids together with solid lipids can lead to the formation of NLC. In the present study, Olivem 1000 (cetearyl olivate, sorbitan olivate) and tea seed oil were used as a solid lipid and a liquid lipid, respectively. A non-ionic surfactant, Tween 80, a cationic surfactant, Varisoft 442, and a combination of Tween 80 and Varisoft 442 were used to obtain three different NLC formulations, i.e., NLC-T, NLC-V, and NLC-C, respectively. NLC-T is expected to be used in skin care products (both facial and body care), whereas NLC-V can provide antimicrobial activity from Varisoft 442, which is a cationic surfactant, and is expected to be formulated for hair care products such as shampoo, conditioner, and hair-treatment. Therefore, NLC-C as comprised of the two surfactants is suitable for both hair care and skin care products. It was found that the three NLC formulations could be formed by using the desirable amount of the surfactant systems in a suitable proportion of Olivem 1000 and tea seed oil. In addition, the surfactant systems displayed an important role on the physicochemical properties of the obtained NLC formulations as described below.

### 3.3. Characterization of NLC

**Figure 3:**
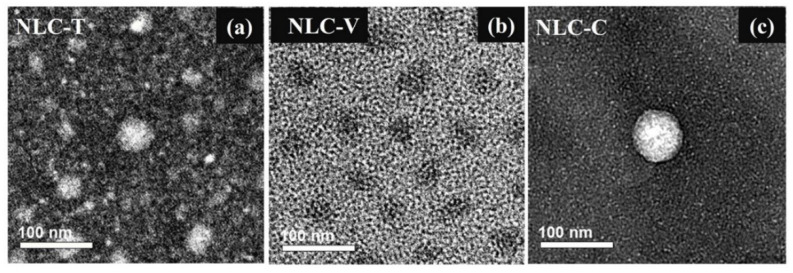
TEM images of NLC-T (**a**), NLC-V (**b**), and NLC-C (**c**). The bars are 100 nm.

**Figure 4:**
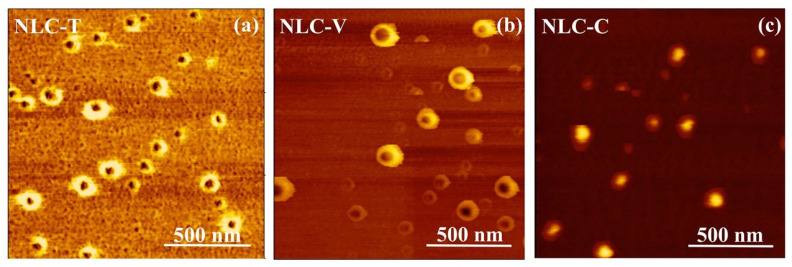
The tapping mode AFM phase images of NLC-T (**a**), NLC-V (**b**), and NLC-C (**c**). The bars are 500 nm.

The prepared NLC particles were characterized using TEM, AFM, and PCS. Investigation by TEM and AFM demonstrate that the NLC-T, NLC-V, and NLC-C particles are spherical in shape, as shown in Figure 3 and Figure 4, respectively. From the TEM images, the average size of NLC-T particles is approximately 90–100 nm, with a relatively wide size distribution, whereas NLC-V particles show a rather uniform size of approximately 90–100 nm. The size of NLC-C particles is also similar to the NLC-T and NLC-V particles, with approximately 80–100 nm. It is clearly seen that all NLC nanoparticles are in the acceptable nanoscale range of the desired formulation. The AFM technique has been widely applied to obtain size, shape, and surface morphological information of nanoparticles. AFM can display surface details down to 0.01 nm and create a contrasting and three-dimensional image of the sample. TEM and AFM works on dry samples. The particle size measured by AFM tends to be the same as that measured by TEM. Under AFM, the diameters of NLC-T and NL-V particles are approximately 90–120 nm, whereas that of NLC-C particles is approximately 80–100 nm. 

The particle size obtained from the TEM and AFM images is often inconsistent with the size analysis obtained from PCS. After measuring with PCS, the sizes of NLC-T and NLC-V particle sizes were found to be in the range of 280–430 nm, whereas that of NLC-C particles was approximately 130–290 nm. PCS is a dynamic light scattering (DLS) principal dimensioning instrument. DLS can determine the size distribution profile of small particles in suspension. It has an advantage of being fast and non-invasive; however, it does require low particle concentrations [52]. DLS is an intensity-based technique [53], whereas TEM and AFM are numerical measurements [54], making them fundamentally different. While the samples for DLS are solvated, TEM and AFM work on dry samples under high-vacuum conditions [55]. DLS measures the hydrodynamic radius of the colloidal dispersed particles, whereas TEM provides the projected image of the particles based on how much of the incident electrons were transmitted through the sample. In the TEM and AFM experiments, the NLC samples were dried in a desiccator oven. After drying, the particles were dry and shrunken, and were no longer colloidal in the dispersion. Therefore, the size obtained from TEM and AFM is usually smaller than that obtained from DLS. AFM images of all NLC formulas show dark core particles surrounded by bright shells. Olivem 1000 is a wax composed of cetearyl olivate and sorbitan olivate. This wax is well-arranged with limited imperfection holes [56]. The addition of some oils may distort the formation of lipid arrangement of the wax by incorporating into these holes as a monolayer film and interacting with wax molecules, resulting in an imperfect crystal lattice of the mixtures. This can be explained in that the oil, at first, may spread through the surface of the wax and modify the interfacial tension. Therefore, the residual oil gathers in a completely separate phase from the wax and covers the surface of the wax, leading to the core–shell structure.

### 3.4. Stability of NLC

**Figure 5:**
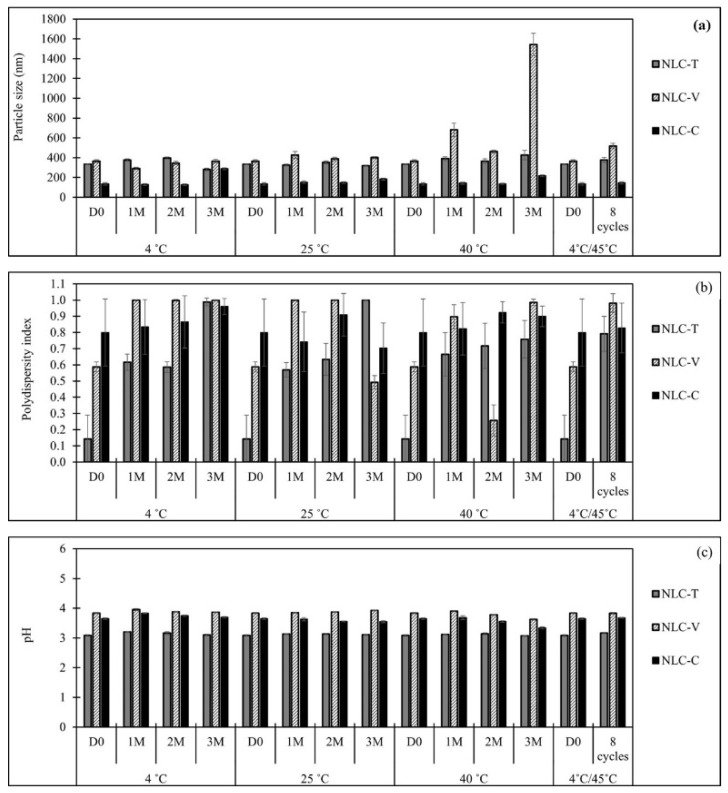
Particle size (**a**), PdI (**b**), and pH values (**c**) of NLC-T, NLC-V, and NLC-C stored at 4 °C, 25 °C, 40 °C for 3 months. D0 = day 0; 1M, 2M, 3M mean 1, 2, 3 months of storage, respectively. Note that 4 °C/45 °C = stored at 4 °C for 48 h and 45 °C for 48 h count as 1 cycle.

For nano-formulations such as NLC, the particle size and size distribution play an important role in the stability of these colloidal dispersions [57]. In this stability study, PCS was used to measure their size and size distribution because the data obtained are more precisely than that from TEM or AFM. The results are shown in Figure 5. It is found that the freshly prepared NLC with a particle size far below 1 µm could be obtained by using high-speed homogenization at 18,000 rpm at 75 °C for 5 min. Stored under different conditions, the prepared NLC particles show different changes. At the end of the 3-month storage period, all NLC samples remained the same pale opaque white as they were at the beginning after fresh preparation. The average diameters of the obtained NLC particles during a storage period are shown in Figure 5a. It is found that the particle size of NLC-T particles remains in the range of 280–430 nm and that of NLC-C particles remain in the range of 130–290 nm over 3 months. These results indicate that both NLC particles possess good long-term stability. The exception is NLC-V kept at 40 °C. Storing at this temperature, NLC-V particles showed significantly larger particle sizes than both NLC-T and NLC-C. The particle size of NLC-V was increased to 680 nm after 1-month storage and became 1550 nm after 3-month storage. However, under fluctuated conditions (4 °C/45 °C), the size of NLC-V particles was only slightly larger.

Polydispersity index (PdI) provides information about the width of particle size distribution. It is also called the heterogeneity index, which describes the degree of non-uniformity of particle size distribution. The PdI value ranges from 0 to 1. The value near zero indicates more homogeneous distribution. Danaei et al. [58] stated that the endocytosis-dependent cellular uptake was influenced by controlling the particle size and PdI. Moreover, they can affect the product performance, bulk properties, stability, and physical appearance of the finish pharmaceutical grade products. Therefore, they are the key-importance parameters for the effective clinical applications of nanocarrier formulations. In this study, the size distribution was reported in terms of PdI, as shown in Figure 5b. The PdI of NLC particles is quite high, indicating that the size distribution is in a wide range. In general, NLC suspensions contain both small and large particles, which can lead to further aggregation and agglomeration of particles. However, NLC-T and NLC-C remained the same size and still had good long-term stability. Among the three NLC formulations, NLC-V showed the highest PdI. This might lead to the growth of NLC-V particles more easily than the others.

Although one of the most popular uses of zeta potential (ZP) data is to relate them with colloid stability, it does not reflect the entire picture. According to the most widely accepted theory of DLVO (named after inventors Derjaguin, Landau, Verwey, and Overbeek), colloid stability depends on the sum of van der Waals attraction and electrostatic repulsion due to the electric double layer [59]. While ZP provides information on the electrostatic repulsive forces, it does not provide any insight on the attractive van der Waals forces. Therefore, it is not uncommon to come across stable colloids with low ZP and vice versa [60]. Hence, we left zeta potential analysis out of the scope of our study.

The pH of all formulations as measured by a pH meter (Mettler Toledo) is shown in Figure 5c. The pH value is used for monitoring the stability of NLC formulations. It can measure the occurrence of chemical reactions which indicate the quality of the NLC formulations. Hydrolysis of the fatty acid esters can generate free fatty acids, which may decrease pH values [61]. In this study, the pH of NLC-T, NLC-V, and NLC-C stayed constant at 3, 4, and 3.7, respectively. No significant change in pH during 3-month storage and the accelerated condition was observed.

### 3.5. Effects of NLC on HFDP Cells

**Figure 6:**
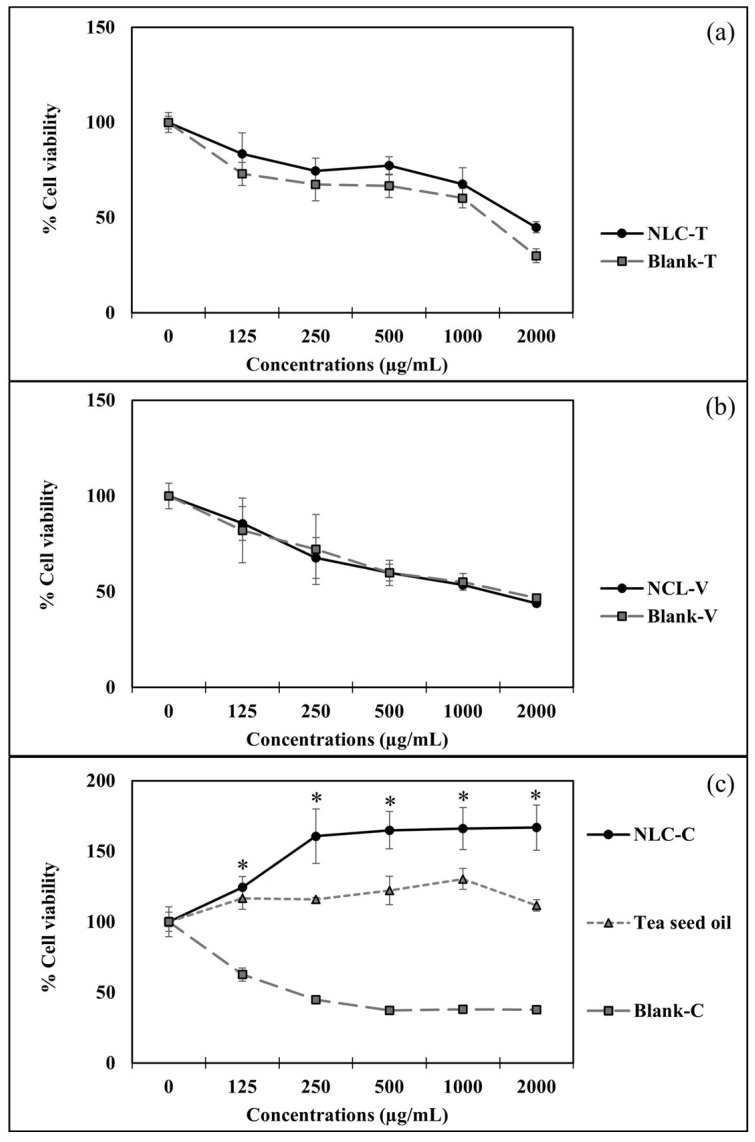
Percentage of cell viability of NLC-T and its blank (**a**), NLC-V and its blank (**b**), and NLC-C and its blank in comparison with tea seed oil (**c**) at the oil concentrations of 0, 125, 500, 1000, and 2000 µg/mL (* indicates statistical significance at *p* < 0.05).

**Figure 7:**
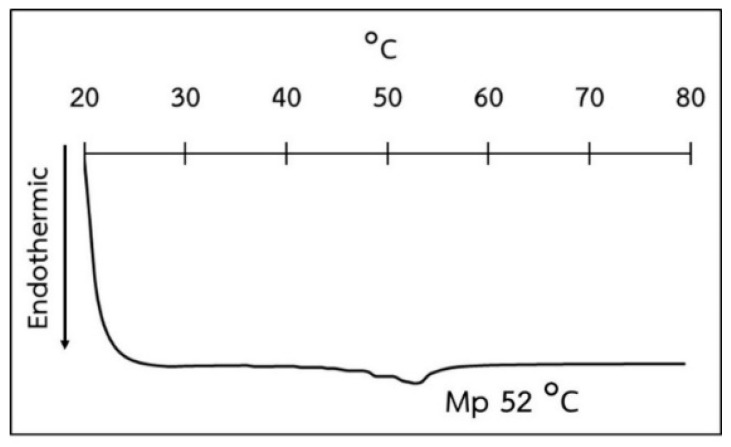
DSC thermogram of NLC-C.

The HFDP cell proliferation profiles due to NLC-T, NLC-V, and NLC-C are shown in Figure 6. It was found that NLC-V, as shown in Figure 6a, and NLC-T, as shown in Figure 6b, did not stimulate the growth of HFDP cells. Moreover, they were highly toxic to the follicle cells. After treating HFDP cells with both types of NLC, the percentage of cell viability was reduced to 20–50% across all ranges of concentrations tested compared to untreated controls. In addition, the percentage of viable cells treated with these was not different from their respective blanks (Blank-V and Blank-T). It was considered that the cytotoxicity of these two NLC formulations was due to the high concentrations of each surfactant reaching toxic levels. Figure 7c shows that the NLC-C was not toxic to HFDP cells. It shows a significant increase in cell proliferation at all tested concentrations. NLC-C is considered to be significantly safer than NLC-V and NLC-T. This may be due to the combination of surfactants, reducing by half the amount of each surfactant and not reaching each surfactant’s toxic level.

NLC-C was compared to its blank (Blank-C). It could stimulate cell proliferation approximately by 120–150% at the concentration range of 125–2000 µg/mL. In contrast, Blank-C was cytotoxic, with a 50% reduction in cell survival at concentrations of 500–2000 µg/mL compared to the untreated control group. Considering the components in each blank sample, they were composed of Tween80 and/or Varisoft442 as a surfactant. These surfactants can cause cell death. Tea seed oil was not only non-toxic to HFDP cells, but also effective in protecting HFDP cells from the toxicity of these surfactants. NLC-V and NLC-T showed high toxicity to HFDP cells even when tea seed oil was entrapped inside. This may be dependent on the physicochemical properties of the nanoparticles. The particle size of NLC-V (370–1550 nm) and NLC-T (280–430 nm) are significantly larger than NLC-C (130–290 nm). NLC-C composed of the smallest particles can penetrate effectively into the cells and release tea seed oil to protect the cells. In addition, NLC-C displayed 20–30% significantly higher ability to stimulate HFDP cell growth than the pure tea seed oil at a concentration in the range of 250–2000 µg/mL. This result shows that the stimulated HFDP cell growth activity of NLC-C is significantly higher than the pure oil and confirms the potential of NLC-C formulation to improve the penetration of tea seed oil into the cells.

### 3.6. Thermal Behavior of NLC

Thermal behavior is one of the important characteristics of samples that shows some physical changes after receiving thermal energy. This can be investigated by using several instruments such as DSC, thermogravimetric analyzers, etc. In the present study, DSC was used to investigate the thermal behavior of the obtained NLC formulations. As NLC is a drug-delivery system composed of both solid and liquid lipids as the core matrix, therefore, a physical state in the form of solid is necessary for the NLC lipid matrix. The DSC thermogram of NLC-C is shown in Figure 7. It is found that the melting point of NLC-C is 52 ± 0.02 °C. The DSC study also reveals that NLC-C possesses melting point over 37–40 °C, confirming a solid state at both skin temperature and room temperature. According to this, NLC-C should be the most suitable formulation. Based on the DSC result and together with the above results showing the smallest size, most stability, and most effectiveness on cell growth stimulation of NLC-C, this formulation was selected for further investigation of drug loading efficiency of the NLC.

### 3.7. Drug Loading Efficiency

In the present study, the amount of tea seed oil loaded in the NLC is expressed as the percentage of drug EE, and it was evaluated immediately after the NLC was produced. It is found that the EE of tea seed oil in the NLC-C particles is 96.26 ± 2.26%. Lipophilic drugs result in high EE of NLC because they are highly associated with lipid phase [62]. The result of this study that shows high quantitative encapsulation of tea seed oil is due to the high lipophilicity of the oil in combination with its low aqueous solubility. This is as oleic acid is one of the active ingredients of tea seed oil. It is found that the EE of oleic acid loading in the NLC-C particles is 17.87 ± 0.35%. GC analysis indicates that no tea seed oil was present in the supernatant aqueous phase of the NLC-C formulation.

### 3.8. Texture Analysis

**Figure 8:**
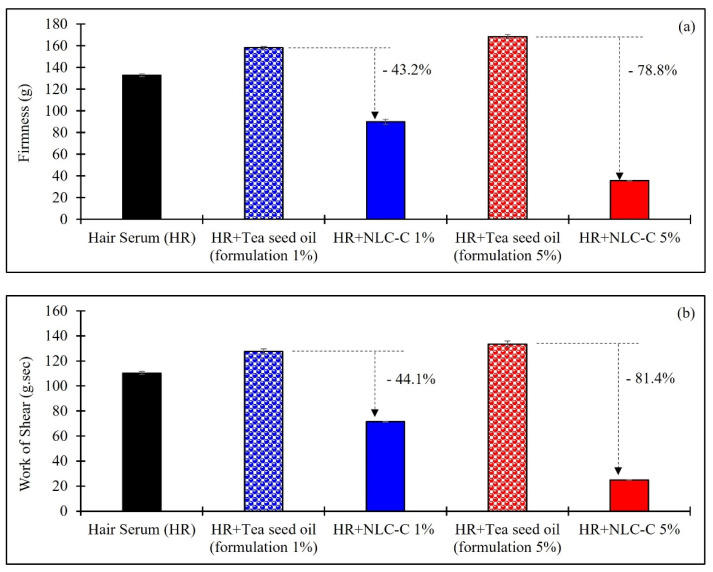
Firmness (**a**), work of shear (**b**) of various samples using a texture analyzer.

Investigation of texture indicates the spreadability of the formulation. Spreadability or spreading capacity provides useful information about perceptual attributes of hair serum. Spreadability improves as the firmness reduces. At the start of the test, the conical probe proceeded and penetrated the samples. During the test, the force was increased until reaching the maximum point of penetration depth. This force can identify the “firmness” of the formulation at the specified depth. The tighter sample also showed a corresponding larger area, which represented the total amount of force required to shear the sample. Both values were used to rank the samples in order of spreadability. When the prepared hair serum containing NLC-C was compared with that containing intact tea seed oil at the same oil concentrations, the firmness of hair serum containing NLC-C particles at 1% and 5% were reduced by 43.2% and 78.8%, respectively, as shown in Figure 8a. Moreover, the spreading efficiency of hair serums containing NLC-C particles at 1% and 5% were improved by 44.1% and 81.4% reduction in shearing force, respectively, as shown in Figure 8b. It was found that hair serums containing NLC-C particles were more dispersed than formulations containing the intact oil. Tea seed oil has an oily consistency, stickiness, and gooey feel. NLC-C particles reduced the firmness of the hair serum; therefore, less shearing force was required. Therefore, tea seed oil in NLC-C particles solves the problems of viscosity, stickiness, and gooeyness from the use of intact oil. NLC can relieve the oiliness and stickiness of hair serums.

## 4. Conclusions

In this study, antioxidant activity and the hair growth-stimulating effect of tea seed oil is confirmed. Three types of tea seed oil-loaded NLC—NLC-T, NLC-V, and NLC-C—were successfully formulated. Among them, NLC-C is the most suitable formulation for hair care product. It is a milky white liquid containing spherical particles of approximately 138–120 nm in diameter. After storing at 4 °C, 25 °C, and 40 °C for 3 months, the NLC-C particles have good stability, with the same size as the fresh preparation. DSC confirmed the solid state of NLC-C at both body and room temperatures. NLC-C also shows high efficiency on entrapment of tea seed oil and high effectiveness in protecting HFDP cells. NLC-C is not toxic to HFDP cells and can enhance the hair growth-stimulating effect of tea seed oil. These positive findings indicate the potential for value-added NLC development using tea seed oil, which is an ingredient extracted from natural sources, as alternative to synthetic drugs. NLC-C particles can effectively reduce the firmness of the hair serum, resulting in a more easily spreadable hair serum. Hair serum containing NLC-C particles ameliorates the stickiness, oiliness, and gooeyness of tea seed oil after application.

## Figures and Tables

**Table 1:** Composition of three NLC formulations containing tea seed oil.

Ingredient	NLC-T	NLC-V	NLC-C
Olivem 1000	2 g	2 g	2 g
Tea seed oil	8 g	8 g	8 g
Tween 80	4 g	-	2 g
Varisoft 442	-	5 g	2.5 g
DI water	86 g	85 g	85.5 g
Total	100 g	100 g	100 g

## Data Availability

All data available are reported in the article.
